# Nicotine exposure of male mice produces behavioral impairment in multiple generations of descendants

**DOI:** 10.1371/journal.pbio.2006497

**Published:** 2018-10-16

**Authors:** Deirdre M. McCarthy, Thomas J. Morgan, Sarah E. Lowe, Matthew J. Williamson, Thomas J. Spencer, Joseph Biederman, Pradeep G. Bhide

**Affiliations:** 1 Center for Brain Repair, Department of Biomedical Sciences, Florida State University College of Medicine, Tallahassee, Florida, United States of America; 2 Pediatric Psychopharmacology, Department of Psychiatry, Massachusetts General Hospital, Harvard Medical School, Boston, Massachusetts, United States of America; Icahn School of Medicine at Mount Sinai, United States of America

## Abstract

Use of tobacco products is injurious to health in men and women. However, tobacco use by pregnant women receives greater scrutiny because it can also compromise the health of future generations. More men smoke cigarettes than women. Yet the impact of nicotine use by men upon their descendants has not been as widely scrutinized. We exposed male C57BL/6 mice to nicotine (200 μg/mL in drinking water) for 12 wk and bred the mice with drug-naïve females to produce the F1 generation. Male and female F1 mice were bred with drug-naïve partners to produce the F2 generation. We analyzed spontaneous locomotor activity, working memory, attention, and reversal learning in male and female F1 and F2 mice. Both male and female F1 mice derived from the nicotine-exposed males showed significant increases in spontaneous locomotor activity and significant deficits in reversal learning. The male F1 mice also showed significant deficits in attention, brain monoamine content, and dopamine receptor mRNA expression. Examination of the F2 generation showed that male F2 mice derived from paternally nicotine-exposed female F1 mice had significant deficits in reversal learning. Analysis of epigenetic changes in the spermatozoa of the nicotine-exposed male founders (F0) showed significant changes in global DNA methylation and DNA methylation at promoter regions of the dopamine D2 receptor gene. Our findings show that nicotine exposure of male mice produces behavioral changes in multiple generations of descendants. Nicotine-induced changes in spermatozoal DNA methylation are a plausible mechanism for the transgenerational transmission of the phenotypes. These findings underscore the need to enlarge the current focus of research and public policy targeting nicotine exposure of pregnant mothers by a more equitable focus on nicotine exposure of the mother and the father.

## Author summary

Use of tobacco products is a major public health concern throughout the world. Cigarette smoking by pregnant women receives significant attention by scientific, public health, and public policy experts because it poses health risks for the mother and her children. Although more men smoke cigarettes than women, the health consequences of paternal smoking for their descendants are much less explored. Using a mouse model, we show that the offspring of nicotine-exposed males have hyperactivity, attention deficit, and cognitive inflexibility. These behavioral phenotypes are associated with attention deficit hyperactivity disorder (ADHD) and autism spectrum disorder in humans. Cognitive inflexibility persists into the third (F2) generation. The neurotransmitters dopamine and noradrenaline and their receptors, critically implicated in neurodevelopmental disorders, are also altered in the offspring’s brains. The nicotine-exposed males show significant alterations in spermatozoal DNA methylation patterns, especially at dopamine receptor gene promoter regions, implicating epigenetic modification of germ cell DNA as a mechanism for the transgenerational transmission of the behavioral and neurotransmitter phenotypes. The impact of nicotine on germ cells and the neurobehavioral impairments in multiple subsequent generations call for renewed focus of research and public policy on tobacco use by men and its consequences for their descendants.

## Introduction

Nicotine use by pregnant women is associated with increased risk of behavioral disorders, not only in their children but also in multiple generations of descendants [1–5]. Whereas maternal nicotine use is an undeniable concern, in reality more men smoke cigarettes than women [6, 7]. Studies in human subjects suggest that paternal cigarette smoking adversely impacts attentional control [8] and increases the risk for attention deficit hyperactivity disorder (ADHD) in the offspring [9, 10]. However, human studies cannot fully separate the effects of paternal smoking from those of genetic and environmental factors [8, 9]. For example, ADHD and nicotine addiction are often comorbid, and ADHD tends to run in families, making it difficult to separate the role of paternal ADHD from paternal smoking on behavioral changes observed in the offspring [8, 10]. Therefore, experimental animal models are valuable tools to address the specific role of paternal nicotine exposure [11].

We exposed male mice to nicotine and bred the mice with drug-naïve females to produce the F1 generation. We bred male and female F1 mice to produce the F2 generation. We found that male and female mice in the F1 and F2 generations showed significant impairment in multiple behavioral phenotypes. The F1 generation also showed significant changes in monoamine neurotransmitter signaling mechanisms in the brain. Analysis of spermatozoal DNA from the nicotine-exposed founder males suggested that nicotine-induced epigenetic modification of the DNA may be a plausible mechanism for the transgenerational transmission of the nicotine-induced behavioral and neurotransmitter phenotypes.

## Results

**Fig 1:**
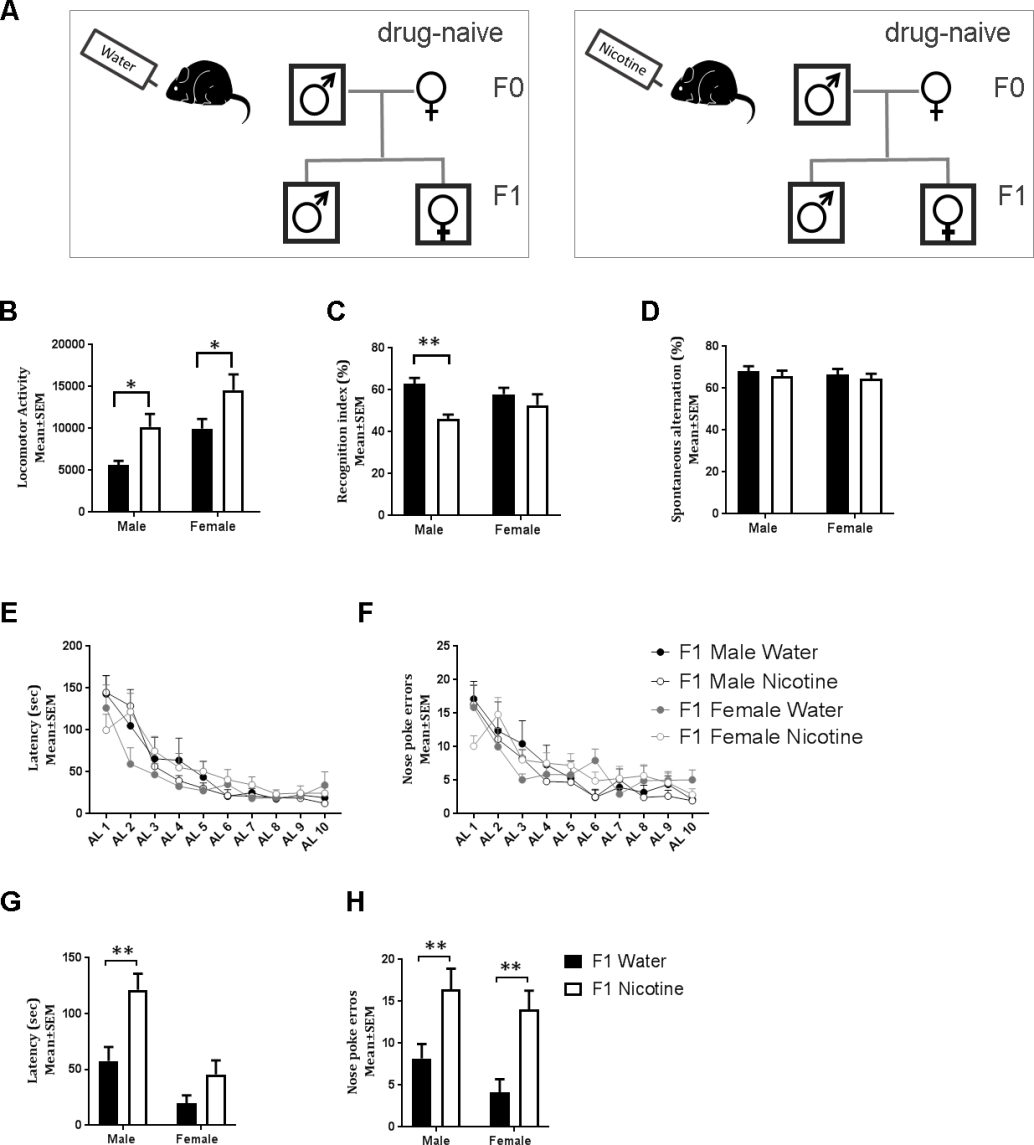
Paternal nicotine exposure paradigm and behavioral phenotypes in F1 male and female mice. (A) Paternal exposure to nicotine in drinking water and production of F1 mice. (B) Spontaneous locomotor activity was measured in F1 mice over a 12-h period (19:00 to 07:00 h; lights-off period; F1 water male *n* = 18; F1 nicotine male *n* = 12; F1 water female *n* = 13; F1 female nicotine *n* = 11). (C) Attention was assayed using recognition index in an object-based attention test (F1 water male *n* = 8; F1 nicotine male *n* = 7; F1 water female *n* = 9; F1 female nicotine *n* = 7), and (D) spatial working memory was assayed using spontaneous alternation index in a Y-maze (F1 water male *n* = 12; F1 nicotine male *n* = 13; F1 water female *n* = 10; F1 female nicotine *n* = 10). A Barnes Maze was used to measure acquisition learning and reversal learning based on latency to escape (panel E and G) and nose poke errors (panel F and H) (F1 water male *n* = 8; F1 nicotine male *n* = 11; F1 water female *n* = 8; F1 female nicotine *n* = 11). Data were analyzed by two-way ANOVA. When main effect or interaction was significant, the ANOVA was followed by Bonferroni post hoc test. Asterisks for post hoc comparisons **p < 0.01 and *p < 0.05 (S1 Data).

We exposed one group of male C57BL/6 mice to drinking water containing 200 μg/mL nicotine (Sigma, N3876) and another group of control male mice to plain drinking water. Following 12 wk of such exposures, and while the exposures were ongoing, the males were bred with drug-naïve female mice to produce the F1 generation (Fig 1A). The nicotine-exposed males had a serum cotinine (primary metabolite of nicotine) level of 77.18 ± 3.06 ng/mL, whereas cotinine was not detectable in the serum of water-exposed control mice.

There was no significant difference in the daily water consumption between the nicotine-exposed and plain-drinking-water–exposed groups (mean ± SEM; mL/d: water: 5.5 ± 0.4; nicotine: 5.4 ± 0.6). When we examined body weights of mice in the 2 groups, there was no significant main effect of treatment (F _[1, 40]_ = 0.01; *p* > 0.05) or treatment × time interaction (F _[4, 40]_ = 18.0; *p* > 0.05) on body weight gain. The effect of time was significant (F _[4, 40]_ = 0.79; *p* < 0.0001), consistent with body weight gain over time expected in both groups of mice.

We analyzed the length of gestation, litter size at birth, and weight gain of the F1 offspring. There was no significant difference in any of these measures between the offspring from the water or nicotine exposure groups (Table 1). Time of acquisition of developmental milestones such as ear detachment, fur appearance, and eye opening were also not significantly different (Table 1).

**Table 1:** Litter metrics and developmental miletones for F1 Offspring.

	Paternal Treatment Group	
F1 Metrics	Water	Nicotine
**Length of gestation (d)**	18.5 ± 0.5	18.4 ± 0.4
**Litter size**	6.3 ± 0.4	7.5 ± 0.4
**Postnatal weight (g) on:**		
P0	1.3 ± 0.1	1.4 ± 0.1
P7	3.9 ± 0.5	3.9 ± 0.2
P14	5.8 ± 0.5	6.3 ± 0.7
P21	9.0 ± 1.1	10.4 ± 0.6
**Ear detachment (P)**	4.4 ± 0.3	4.0 ± 0.4
**Fur appearance (P)**	4.0 ± 0.0	4.8 ± 0.5
**Eye opening (P)**	13.4 ± 0.3	13.3 ± 0.3

**Abbreviation:** P, postnatal day.

Since nicotine exposure of pregnant dams produces hyperactivity in their offspring [4, 5], we analyzed spontaneous locomotor activity in postnatal day (P) 60 F1 mice over a 12-h period (19:00 to 07:00 h), which was the dark phase of the light-dark cycle when the mice are naturally more active. A two-way ANOVA revealed significant main effects of treatment (F _[1, 50]_ = 13.68; *p <* 0.001) and sex (F _[1, 450]_ = 12.63; *p <* 0.001) but no significant effect of treatment × sex interaction (F _[1,50]_ = 0.0006; *p* > 0.05). Bonferroni multiple comparisons test revealed that the locomotor activity was significantly increased in male and female F1 mice in the paternal nicotine exposure group (Fig 1B and S1 Data) (post hoc: male: t = 2.730; df = 50; *p <* 0.05; female: t = 2.516; df = 50; *p <* 0.05).

Preclinical studies [4, 5, 12–16] and clinical studies [3, 17–20] show that prenatal nicotine exposure produces significant attention deficits. Therefore, we used an object-based attention test [13, 21, 22] to examine the effects of paternal nicotine exposure on attention in the F1 mice. Paternal treatment produced a significant main effect on attention (two-way ANOVA; treatment: F _[1, 35]_ = 9.23; *p <* 0.01). Neither the effect of sex nor the treatment × sex interaction was significant (two-way ANOVA; sex: F _[1, 35]_ = 0.0451; *p* > 0.05; interaction: F _[1, 35]_ = 2.531; *p* > 0.05). Bonferroni multiple comparisons test revealed significant attention deficit in the male (t = 3.148; df = 35; *p <* 0.01) but not female offspring (t = 1.069; df = 35; *p* > 0.05) derived from the nicotine-exposed fathers (Fig 1C and S1 Data).

Working memory and cognitive flexibility are components of executive function [23–27] impacted by drug exposure [28, 29]. We have previously reported that nicotine or cocaine exposure of pregnant mice impairs working memory and cognitive flexibility in the offspring [22, 30]. We analyzed spatial working memory in F1 mice using the Y-maze. There was no significant effect of paternal treatment, sex, or treatment × sex interaction (two-way ANOVA; treatment: F _[1, 41]_ = 0.6889; *p* > 0.05; sex: F _[1, 41]_ = 0.265; *p* > 0.05; interaction F _[1, 41]_ = 0.001; *p* > 0.05; Fig 1D and S1 Data). Next, we examined cognitive flexibility using a Barnes Maze [31–33]. Latency to escape and number of nose-poke errors made in the process of escape were quantified. Over the 10-d period of acquisition learning, paternal treatment did not produce significant effects on either measure (repeated-measures ANOVA; treatment: F _[3, 34]_ = 0.6614; *p* > 0.05 (latency); F _(3, 34)_ = 0.8189, *p* > 0.05 (nose-poke errors); Fig 1E and 1F and S1 Data). Because all mice learned the task equally well, as expected, there was a significant main effect of time (i.e., day of learning) in both analytical measures (latency to escape: F _[9, 306]_ = 37.06; *p <* 0.001; number of nose poke errors: F _[9, 306]_ = 22.17; *p <* 0.001). Treatment × day interaction was not significant (latency: F _[27, 306]_ = 1.328; *p* > 0.05; errors: F _[27, 306]_ = 1.374, *p* > 0.05). However, upon reversal, there was a significant main effect of paternal treatment on latency to escape (two-way ANOVA; F _[1, 34]_ = 12.26; *p* < 0.005) and the number of nose poke errors (two-way ANOVA; F _[1, 34]_ = 16.78; *p* < 0.001). There was a significant main effect of sex on latency (two-way ANOVA; F _[1, 34]_ = 19.57; *p* < 0.001) but not on the number of nose poke errors (two-way ANOVA; F _(1, 34)_ = 2.105; *p* > 0.05). Bonferroni multiple comparisons test revealed that paternally nicotine-exposed male mice showed significant increases in the latency to escape (t = 3.511; df = 34; *p* < 0.05; Fig 1G and S1 Data) as well as nose-poke errors (t = 2.625; df = 34; *p* < 0.05; Fig 1H and S1 Data). Paternally nicotine-exposed female mice had a significant increase in nose-poke errors (t = 3.168; df = 34; *p* < 0.01; Fig 1H and S1 Data). Thus, both male and female mice derived from nicotine-exposed sires showed significant reversal learning deficits.

**Fig 2:**
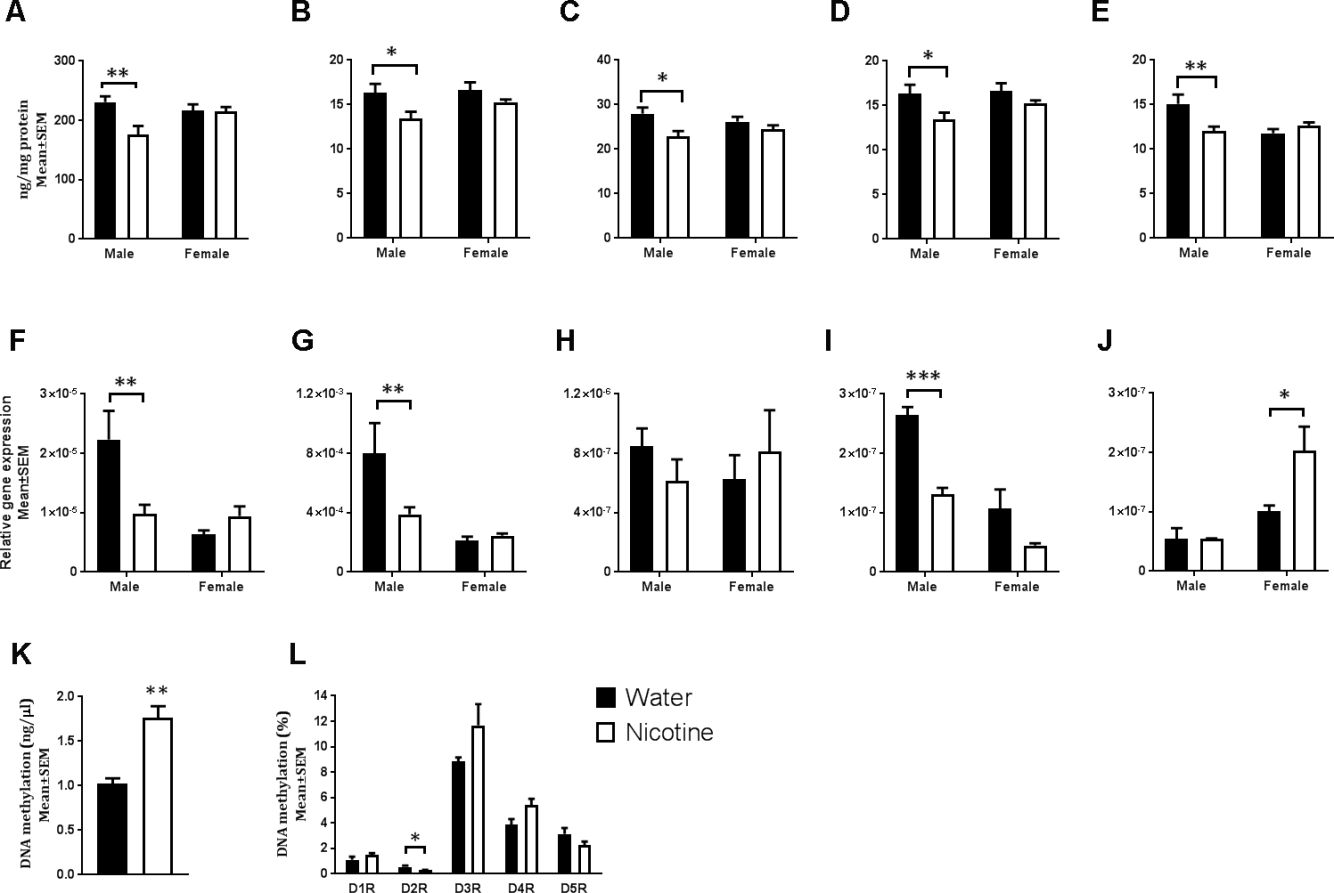
Tissue neurotransmitter content, striatal gene expression, and methylation of spermatozoal DNA. (A) Dopamine and its metabolites (B) DOPAC, (C) HVA, and (D) 3-MT in the striatum and (E) noradrenaline in the frontal cortex were measured (F1 water male *n* = 6; F1 nicotine male *n* = 6; F1 water female *n* = 6; F1 female nicotine *n* = 6). Striatal dopamine D1 (panel F), D2 (panel G), D3 (panel H), D4 (panel I) and D5 (panel J) receptor mRNA expression (F1 water male *n* = 3–5; F1 nicotine male *n* = 4–5; F1 water female *n* = 4; F1 female nicotine *n* = 4). Data were analyzed by two-way ANOVA. When main effect or interaction was significant, the ANOVA was followed by Bonferroni post hoc test. Asterisks for post hoc comparisons ***p < 0.001, **p < 0.01, *p < 0.05. Global DNA methylation (panel K; F0 water *n* = 4; F0 nicotine *n* = 4) and methylation of dopamine receptor promoter regions (panel L; F0 water *n* = 3–4; F0 nicotine *n* = 4–5) in spermatozoa of F0 founders was analyzed by Student unpaired *t* test, **p < 0.01, *p < 0.05 (panel K–L); S2 Data. 3-MT, 3-methoxytyramine; DOPAC, 3,4-dihydroxyphenylacetic acid; HVA, homovanillic acid.

Monoamine signaling in the basal ganglia and frontal cortex regulates motor and cognitive functions [23, 28]. Because paternal nicotine exposure produced hyperactivity, attention deficit, and reversal learning deficit, we examined whether the paternally nicotine-exposed mice showed alterations in monoamine neurotransmitter signaling mechanisms. We analyzed tissue content of dopamine, noradrenaline, and their metabolites in the frontal cortex, orbitofrontal cortex, and the striatum in the F1 offspring. There was a significant main effect of paternal treatment on striatal tissue content of dopamine (two-way ANOVA; dopamine: F _[1, 20]_ = 6.582; *p <* 0.05) and its metabolites 3,4-dihydroxyphenylacetic acid (DOPAC: F _[1, 20]_ = 7.949; *p <* 0.05), homovanillic acid (HVA: F _[1, 20]_ = 8.522; *p <* 0.01), and 3-methoxytyramine (3-MT: F _[1, 20]_ = 7.949; *p <* 0.05). Bonferroni multiple comparisons test showed that paternally nicotine-exposed male F1 mice showed significant deficits in the tissue content of all four molecules (dopamine: t = 3.542; df = 20; *p <* 0.01; DOPAC: t = 2.722; df = 20; *p <* 0.05; HVA: t = 3.06; df = 20; *p <* 0.05; 3-MT: t = 2.722; df = 20; *p <* 0.05; Fig 2A–2D and S2 Data), whereas the female F1 mice did not (dopamine: t = 0.086; df = 20; *p* > 0.05; DOPAC: t = 1.265; df = 20; *p* > 0.05; HVA: t = 1.069; df = 20; *p* > 0.05; 3-MT: t = 1.265; df = 20; *p* > 0.05; Fig 2A–2D). Paternal treatment did not produce a significant main effect on dopamine or its metabolites in the frontal or orbitofrontal cortices (S1 Table). Although neither paternal treatment nor sex produced significant effects on noradrenaline content in any brain region (S1 Table), there was a significant paternal treatment × sex interaction for noradrenaline content in the frontal cortex (two-way ANOVA; F _[1, 20]_ = 8.638; *p <* 0.01). Bonferroni multiple comparisons test revealed a significant decrease in frontal cortical noradrenaline content in the paternally nicotine-exposed male F1 mice (t = 3.257; df = 20; *p <* 0.01; Fig 2E and S2 Data) and no significant change in the female F1 mice (t = 0.8996; df = 20; *p* > 0.05; S1 Table).

Next, we examined mRNA expression for dopamine receptor genes by quantitative PCR (qPCR) in the striatum and frontal cortex. Paternal treatment had a significant effect on D2 (two-way ANOVA; F _[1, 12]_ = 5.364; *p <* 0.05) and D4 (F _[1, 11]_ = 25.33; *p <* 0.001) receptor mRNA expression, and there was a significant main effect of sex on D1 (two-way ANOVA; F _[1, 12]_ = 13.63; *p <* 0.01) and D5 (F _[1, 11]_ = 17.37; *p <* 0.01) receptor mRNA expression. Bonferroni multiple comparisons test showed that D2 (t = 3.519; df = 12; *p <* 0.01) and D4 (t = 4.682; df = 11; *p <* 0.01) receptor mRNA levels and the D1 receptor mRNA level (t = 3.994; df = 12; *p <* 0.01) were significantly decreased in the striatum of paternally nicotine-exposed male F1 mice (Fig 2G and 2I and S2 Data). On the other hand, the D5 receptor mRNA expression was significantly higher (t = 3.205; df = 11; *p* < 0.05) in the striatum of paternally nicotine exposed F1 female F1 mice (Fig 2F and 2J and S2 Data). Dopamine D3 receptor mRNA expression was not significantly altered by the paternal treatment (F _[1, 13]_ = 1.36; *p* > 0.05) or sex (F _[1, 13]_ = 0.003; *p* > 0.05) (Fig 2H and S2 Data). There was no significant effect of paternal treatment, sex, or treatment × sex interaction in dopamine receptor mRNA expression in the frontal cortex (S2 Table).

Because the F1 mice were not exposed to nicotine at any time before or after birth, the behavioral and neurotransmitter phenotypes in these mice are likely inherited from the founder generation. The F1 phenotypes were not consistent with Mendelian inheritance, suggesting nicotine-induced epigenetic modification of the father’s spermatozoal DNA or histones as a plausible mechanism of transgenerational transmission [34]. In germ cells, the majority of histones are replaced with protamines during development [35, 36][37]. Therefore, epigenetic modification of the DNA, rather than histones, appeared more likely. Because nicotine is known to alter DNA methylation in somatic cells [38, 39], we focused on DNA methylation. We collected spermatozoa samples from the cauda epididymis using a double swim assay [40]. Analysis of total numbers of sperm showed that nicotine exposure did not produce significant changes in this parameter (mean ± SEM, number/mL: water: 1.1 ± 0.2 × 10^6^; nicotine: 1.2 ± 0.1 × 10^6^).

We isolated spermatozoal DNA from nicotine-exposed and control males and analyzed global DNA methylation as well as methylation at promoter regions of the dopamine receptor genes. Global DNA methylation was significantly increased by the nicotine exposure (t = 5.015; df = 6; *p* < 0.01; Fig 2K and S2 Data), and DNA methylation was significantly decreased at the dopamine D2 receptor promoter region (t = 3.409; df = 6; *p* < 0.05; Fig 2L and S2 Data). There was no significant change in the methylation status of promoters of the other dopamine receptor genes (Fig 2L).

**Fig 3:**
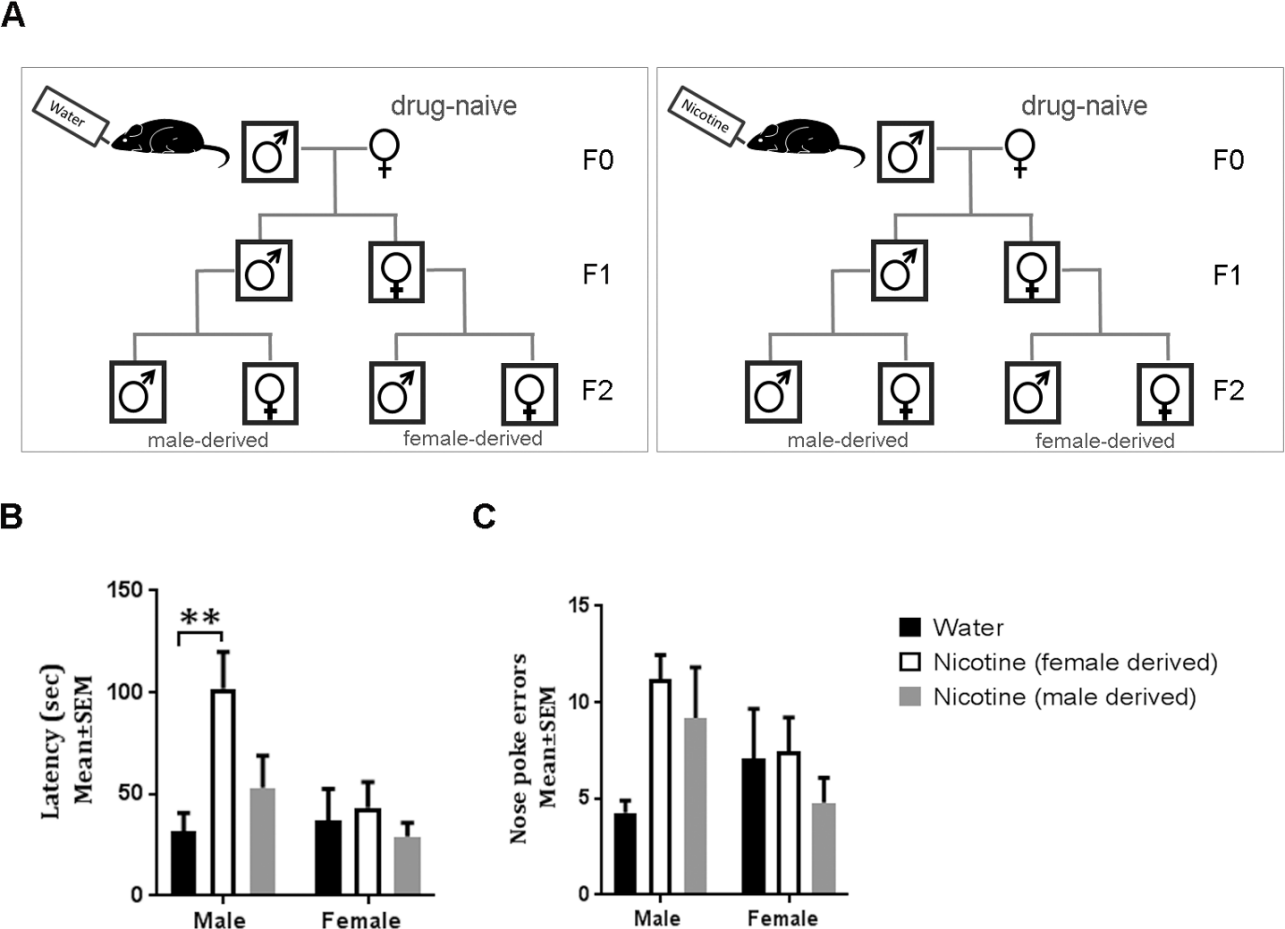
Multigenerational transmission of reversal learning phenotype. (A) Generation of F2 mice from male and female F1 mice. A Barnes maze was used to assay reversal learning based on (B) latency to escape or (C) nose poke errors (F2 water male *n* = 10; F2 female-derived nicotine male *n* = 11; F2 male-derived nicotine male *n* = 5; F2 water female *n* = 9; F2 female-derived nicotine female *n* = 9; F2 male-derived nicotine female *n* = 5). Data were analyzed by two-way ANOVA. When main effect or interaction was significant, the ANOVA was followed by Dunnett post hoc test. Asterisks **p < 0.01 represent the post hoc analysis; S4 Data.

To examine whether behavioral phenotypes observed in the F1 generation persisted beyond the F1 generation, we produced F2 mice from male and female F1 mice (Fig 3A). We analyzed spontaneous locomotor activity, attention, spatial working memory, and reversal learning in the F2 mice. There were no significant changes in spontaneous locomotor activity, object-based attention, or working memory in male or female F2 mice whether derived from male or female F1 founder (S1 Fig and S3 Data). However, paternal treatment (of the founder or F0 generation) had a significant main effect on latency to escape during reversal learning in the F2 generation (two-way ANOVA; treatment: F _(2,42)_ = 4.354, *p <* 0.05). Because comparisons were made against a single group of F2 control mice (derived from F1 male or female mice descending from F0 male mice that were exposed to plain drinking water), we used Dunnet’s multiple comparisons test for analysis of the differences among the groups [22]. Male F2 mice derived from female F1 founders showed significant deficits in reversal learning when latency to find the escape hole (t = 3.838; df = 42; *p <* 0.001; Fig 3B and S4 Data) was considered as the parameter. Male F2 mice derived from male F1 founders and female F2 mice derived from male or female F1 founders did not show significant changes in reversal learning (Fig 3B and 3C and S4 Data).

## Discussion

We show that paternal nicotine exposure is associated with sex-dependent changes in behavioral phenotypes, monoamine content in the striatum and frontal cortex, and striatal dopamine receptor mRNA expression. The behavioral changes are apparent in F1 and F2 generations but not in the F0 generation. Nicotine-induced spermatozoal DNA methylation at dopamine receptor promoter regions may be a plausible epigenetic mechanism for transgenerational transmission of the effects of the paternal nicotine exposure.

Recent evidence shows that the effects of a variety of environmental stimuli, including stress, hormones, drugs of abuse, nutritional deprivation, and psychological trauma, are heritable, in some instances across multiple generations [5, 11, 41–45]. A first step toward defining the molecular mechanisms of transgenerational transmission of environment-induced phenotypes is to identify changes in the germ cell DNA of the exposed generation [11, 45, 46]. Nicotine is known to produce epigenetic modification of DNA in germ cells and somatic cells [11, 38, 47]. Consistent with those findings, our present data also show that nicotine alters DNA methylation in the spermatozoa. The precise mechanisms mediating the nicotine-induced epigenetic changes are unclear and likely include the action of noncoding RNAs such as microRNAs (miRNAs) as well as histone modification besides the DNA methylation demonstrated here [36, 37, 48]. In fact, a recent study showed that miRNAs can mediate the effects of paternal nicotine exposure on behavioral and molecular phenotypes in the F1 generation [11].

Epigenetic marks on germ cell DNA are erased and reinstated on multiple occasions before and after fertilization, casting some doubt that such labile molecular signatures could be the basis for transgenerational transmission. However, developmentally relevant genes “escape” the global erasure of epigenetic marks during the embryonic period, and these “escapees” remain plausible candidates for transgenerational transmission [49]. The dopamine receptor genes are key components of developmental pathways such as neurogenesis, neuronal migration, and differentiation regulated by dopamine [50, 51]. Therefore, nicotine-induced changes in DNA methylation at dopamine receptor promoter regions could remain stable and form the basis of transgenerational transmission of nicotine’s effects at least in this model of paternal nicotine exposure. Another example of nonlabile epigenetic modification is DNA methylation produced by psychoactive drugs during adolescence, which lasts into adulthood and can be inherited by the offspring [52].

A link between the reduced striatal dopamine D2 and D4 receptor mRNA expression produced by the paternal nicotine exposure and the phenotypes of attention deficit and hyperactivity in the F1 mice is consistent with reports of association between polymorphisms in the D4 receptor gene and ADHD, and the role of D2 receptor in hyperactivity in animal models of ADHD [53–55]. This raises the possibility that the paternal nicotine exposure mouse model may have significant face validity as an ADHD model. Although we did not find significant changes in dopamine receptor mRNA expression in the frontal cortex, a key brain region implicated in ADHD [24], the changes in striatal D2 and D4 receptor mRNA expression demonstrated here are equally relevant to ADHD because striatal function and cortico-striatal communication critically regulate attentional mechanisms [56, 57]. In the paternally nicotine-exposed females, only the D5 receptor mRNA showed a significant effect in the striatum. The significance of this increase remains uncertain.

The present study directly addresses some of the drawbacks associated with human studies on the effects of paternal nicotine exposure on the offspring. For example, in our earlier study on this topic [10], some of the fathers who smoked cigarettes also had ADHD, making it difficult to separate the independent effects of paternal smoking from paternal ADHD on the offspring. In another study on the effects of paternal nicotine exposure on behavioral phenotypes in the offspring [11], the nicotine-exposed male mice (founder generation) displayed significant behavioral changes, some of which were also seen in their offspring. In the present study, which used nicotine exposure rather than cigarette smoke exposure, the nicotine-exposed founder males (fathers) did not display hyperactivity, attention deficit, or working memory deficit (S2 Fig and S5 Data), suggesting that the behavioral phenotypes in the offspring occurred in the absence of similar phenotypes in their fathers. In other words, the present study offers evidence that the behavioral phenotypes in the offspring can emerge in the absence of similar phenotypes in the fathers, addressing a drawback associated with interpretation of data from the previous human [10] and mouse [11] studies. Moreover, the present study used relatively low levels of nicotine exposure compared to previous studies in mice [11, 58]. Therefore, the present study highlights the potential risk of low levels of nicotine exposure for future generations.

Hyperactivity and attention deficit, phenotypes that had arisen de novo in the F1 generation as a result of the F0 nicotine exposure, were not transmitted to the F2 generation. Only the reversal learning deficit was transmitted from the F1 to the F2 generation. Thus, we observed an “attenuation” of the phenotypes during F1 to F2 transmission. The “attenuation” suggests that at least some of the deleterious effects of the nicotine exposure may be transient. However, repeated exposure of each successive generation might render the phenotypes more permanent and perhaps even endemic to the population.

Finally, the present study analyzed paternal nicotine-exposure–induced phenotypes in both male and female offspring in both F1 and F2 generations. In addition, male and female F1 founders derived from the nicotine-exposed and control groups were used to generate the F2 generation, permitting analysis of the role of sex not only in the F1 and F2 generations but also in the F1 to F2 transmission. We found that the F1 and F2 phenotypes as well as the transmission of the reversal learning deficit from the F1 to the F2 generation were sex dependent. In the F1 generation, hyperactivity and reversal learning deficits occurred in both male and female mice, whereas the attention deficit occurred only in the male mice. The only phenotype in the F2 generation was reversal learning deficit, and it was observed only in the male F2 mice. The F1 to F2 transmission of reversal learning deficit occurred via the maternal but not the paternal line of descent. The maternal transmission is reminiscent of similar observations in our earlier study of transgenerational transmission of hyperactivity following prenatal nicotine exposure [5]. These two studies together suggest potential differences in the vulnerability of male versus female germ cells to nicotine exposure.

Earlier studies that examined preconception paternal [11] or preconception paternal and maternal nicotine exposure [58] reported a number of behavioral phenotypes in the F1 generation. Paternal nicotine exposure produced depression-like and anxiety-like phenotypes as well as reduced locomotor activity and impaired social interaction [11, 58]. Maternal nicotine exposure, on the other hand, produced increased locomotor activity and increased mobility in the forced swim test. The latter is the opposite of the depression-like phenotype produced by the paternal exposure [58]. Following nicotine exposure of both parents, the depression-like phenotype and impaired social interaction were observed. These observations not only suggest that the paternal-only nicotine exposure produces robust changes in multiple behavioral domains, but it also suggests that the phenotypes produced when both parents are exposed to nicotine (namely, depression-like phenotype and impaired social interaction) more closely resemble the phenotypes produced by the paternal-only rather than maternal-only nicotine exposure.

The mechanisms underlying the sex-specific nature of the behavioral and molecular phenotypes observed here remain unclear. Sex differences in nicotine’s effects on the brain and behavior have been described previously [59–62]. Sex differences in hypothalamic-pituitary axis signaling, estrogen receptor signaling, neurotransmitter receptor signaling, and especially dopamine receptor expression are among the candidate mechanisms proposed for sex differences in the effects of nicotine upon the brain and behavior [60, 61, 63, 64]. Nicotine-induced epigenetic modification of the DNA or histones could also contribute to sex-dependent changes reported here. Genetic sex, organizational versus activational influences, imprinted genes, and mitochondrial DNA [65, 66] play a role in the expression of the sex-specific phenotypes. In addition, there are imprint/parent-of-origin effects on transcription at over 1,300 loci and approximately 350 autosomal genes with sex-specific parent-of origin effects in the mouse brain [65, 66].

Cigarette smoke contains over 1,000 chemical substances, many of which can produce changes in DNA methylation [47]. The effects of cigarettes on the brain and behavior are mediated via nicotine’s direct actions at the nicotinic acetylcholine receptor in the developing and mature brain [67, 68]. Nicotine exposure via smokeless tobacco (chewing, snuff or e-cigarettes) is highly prevalent (review in [69]). Equally importantly, the use of e-cigarettes (vaporized nicotine) is increasing, especially among young adults of reproductive age, due to false perceptions of increased safety. Between 2013 and 2014, in just 1 year, the use of e-cigarettes tripled among high school students [70]. Therefore, the nicotine exposure (as opposed to cigarette smoke exposure) paradigm used here has significant ecological and public health validity.

Our 12-wk nicotine exposure paradigm encompassed the entire mouse spermatogenesis cycle [71]. The 12-wk exposure did not produce adverse effects on water or food consumption, body weight gain, or sperm count in the fathers, or on litter size, weight, weight gain, or developmental milestones in the offspring. The mouse strain used here (C56Bl/6) preferentially consumes nicotine when given free choice between plain drinking water and nicotine-containing water [72, 73]. Therefore, stress due to forced exposure to nicotine-laced water is unlikely to be a confounding variable. Finally, our nicotine exposure paradigm did not involve nicotine withdrawal. Paradigms that employ drug self-administration involve withdrawal during breeding, which could be a confounding variable [43].

The behavioral phenotypes, the neurotransmitter and mRNA phenotypes, and the preponderance of the phenotypes in F1 and F2 males observed in the present study are consistent with the clinical presentation, putative neurobiological mechanisms, and rate of diagnosis of ADHD and autism [24, 74, 75]. In addition, 2 of the behavioral phenotypes, namely, attention deficit and cognitive inflexibility (reversal learning deficit), observed in the present study occur in both ADHD and autism [76] [27, 77]. In addition to autism, cognitive inflexibility is a core symptom of schizophrenia, obsessive-compulsive disorder, and anorexia nervosa [78–80]. The higher prevalence of smoking in the 1950s and 1960s compared to today, taken together with our present findings, raises the possibility that nicotine exposure in generations past could be contributing to the rise in the diagnosis of neurobehavioral disabilities such as ADHD and autism in the present generation. Finally, our findings underscore the need to shift the current selective focus of research and public policy on the consequences for future generations of nicotine exposure of the mother to a more equitable focus on nicotine exposure of the mother and the father.

## Materials and methods

### Ethics statement

The studies were approved by the Florida State University Animal Care and Use Committee (protocol number 1714).

### Animals

C57BL/6 mice were housed in the Florida State University Laboratory Animal Resource facility in a temperature- and humidity-controlled environment on a 12-h light-dark cycle with food and water available ad libitum. Male mice (8- to 10-wk-old) were randomly assigned to one of 2 groups: plain drinking water or drinking water containing 200 μg/mL nicotine (Sigma; N3876). Following 12 wk of such exposure and while the exposure was ongoing, the male mice were bred with drug-naive female mice (Fig 1A). The day of birth was designated P0. Litter size was recorded, and pups were weighed on P0, P7, P14, and P21. All litters were standardized to contain 6 to 8 offspring with equal numbers of males and females per litter, and the offspring were weaned about P21. All of the experimental procedures were in full compliance with Florida State University guidelines and the NIH Guide for the Care and Use of Laboratory Animals. From a given litter, no more than 2 to 3 male and female mice were used.

### Behavioral analyses

At the time of weaning, the mice were housed 2 to 4 per cage and were handled by the experimenter for at least 3 min per day for at least 1 wk prior to the beginning of the behavioral analyses at P60. Immediately prior to the commencement of the behavioral testing, mice were habituated to the testing room for at least 30 min. The handling, habituation, and behavioral testing occurred during the lights-off period, when mice are naturally more active. Dim red light was used for ambient illumination and for video recording, with the exception of the spontaneous locomotor activity test, for which video recording was not performed. The spontaneous locomotor activity test spanned a period of 16 h. This included a 2-h long “lights-on” session before the 12-h “lights-off” period. The initial 2-h permitted the mice to habituate to the testing environment. The lights-on sessions were not included in the data analysis. In all behavioral tests, mice from each of the 2 paternal treatment groups (i.e., nicotine or water) were tested concurrently.

### Spontaneous locomotor activity

Locomotor activity was measured in testing chambers equipped with photobeam motion sensors (Photobeam Activity System; San Diego Instruments). The sensors create a 3-dimensional grid (5.4-cm spacing) of infrared beams enveloping the entire cage. Mice were placed individually into testing chambers. As the mouse moves along the x-, y-, or z-axes, the number of breaks in the infrared beams are recorded. Each instance in which the movement of the mouse breaks consecutive beams was scored as an ambulatory event. The photobeam breaks were grouped into hourly activity measurements for statistical analysis. The analysis was conducted over a 12-h period from 19:00 h to 07:00 h (daily lights-off period was between 19:00 h and 07:00 h).

### Y-maze

Spatial working memory was assayed using a custom-built clear Plexiglas Y-maze [22]. Each of the 3 arms of the maze was 35 cm long by 6 cm wide by 10 cm high; distinct visual cues were placed on the walls of each arm and on the walls of the testing room. The mouse was placed at the center of the Y and had free access for exploration of all 3 arms for a period of 6 min. An investigator blinded to the identity of the mouse calculated the number and sequence of arm entries over the 6-min period by analyzing video recordings of the maze exploration. The mouse was considered to have entered an arm only if all 4 limbs entered it. An “alternation” is a set of 3 nonrepeating consecutive arm choices (e.g., ABC, BCA, CBA but not ABB, CCB, BAA, etc.). An alternation index was calculated as follows: number of alternations ÷ (number of entries − 2) × 100.

### Object-based attention test

The rationale behind this test is that mice divide their attention between a familiar and novel object (measured by time spent exploring an object) such that they explore a novel object for a longer duration (i.e., pay more attention) than a familiar object. A mouse with attention deficit is expected to either focus equal attention upon familiar and novel objects or focus less attention on the novel object. Details of the test methodology are described in our earlier publications [22, 81] and are given here in brief. The apparatus consists of a training chamber and a test chamber separated by a sliding door. The test consists of 3 sessions: on day 1, during the habituation session (10 min), mice are individually exposed to each of the 2 chambers (5 min each). On day 2, during the training session (3 min), mice are allowed to explore 5 objects placed in the training chamber. All objects are made from the same wooden material, but each object has a distinct shape (i.e., rectangle, triangle, circle, oval, and octagon). Next, on the same day, the mice are allowed to explore 2 objects of different shapes (e.g., circle and triangle) in the test chamber for 5 min. On day 3, during the test session, the mouse is habituated to the empty training and test chambers for 6 min (3 min in each chamber) and then allowed to explore the same 5 objects used on day 2 in the training chamber for 3 min. Following a 10-s interval, the sliding door is opened, and the mouse is allowed to enter the test chamber to explore 2 objects for 3 min. One of these two objects is randomly selected from the 5 objects that the mouse had explored in the training chamber and is placed in the test chamber in a position analogous to its original position in the training chamber. This object is therefore the familiar object. The second object is a novel object, to which the mouse had never been exposed. A recognition index for the test session is calculated using the formula: TN ÷ (TF + TN) × 100, where TF and TN are time spent exploring the familiar and the novel objects, respectively. We included in the analysis only those mice that spent at least 20 s with both of the objects during the test session.

### Reversal learning

A modified Barnes maze consisting of a circular arena (122-cm diameter and 140-cm height; Med Associates Inc., St. Albans City, VT) with 40 equally spaced holes along the periphery was used. An escape box was positioned under one of the holes to allow the mouse to escape. The position of the escape hole was assigned randomly for each mouse in advance of the test, and the position remained the same throughout the trials for that mouse. Visual cues were placed around the maze to serve as spatial cues. A bright light (150 W) and a fan were positioned above the maze. A starting chamber (metal bowl) held the mice at the center of the maze at the start of each trial.

Mice were tested in squads of 4 in the following 3 phases of testing:

1. Habituation: mice completed two 4-min trials during which they were placed next to the assigned escape hole. The light and fan were turned on for this phase. Usually, the mouse entered the escape hole within the 4-min duration of this phase. If it did not, it was gently guided into it by the experimenter at the end of the 4 min. The light and fan were turned off, and the mouse returned to its home cage.
2. Acquisition learning: mice completed two 4-min trials per day for 10 consecutive days. The experimenter placed the mouse in the starter chamber at the center of the maze. The light and fan were turned on, and the mouse was released from the starter chamber and allowed to freely explore the arena in search of the escape hole. The trial ended when the mouse entered the escape hole. However, at the end of the 4-min trial period, if the mouse had not entered the escape hole, the trial was terminated by gently guiding the mouse into the escape box. The light and fan were turned off. The mouse remained in the escape box for 30 s, and then it was returned to its home cage. The intertrial interval was 15 to 20 min. For each trial, latency (s) to find the escape box and the number of errors made in the process of finding the escape box were recorded. An error occurred when the mouse lowered its head into a nonescape hole. The maze was cleaned with 1.6% Quatracide immediately upon each mouse completing the test to eliminate odor cues.
3. Reversal learning: mice completed two 4-min trials per day for 2 consecutive days. The trials were run as described in the acquisition phase, with one key difference: the escape hole was relocated by 180° (i.e., diagonally opposite the original position). Mice explored the maze to find the new escape hole. A reversal effect was calculated as the difference between RL1 and AL10 measurements, separately for latency and errors. A statistically significant increase in latency and/or errors between the control and experimental groups was interpreted as a reversal learning deficit.

### RNA extraction and reverse transcription

Mice were euthanized on P90 by anesthetic overdose, and the brains were removed. Frontal cortex and dorsal striatum were microdissected from both the hemispheres based on anatomical landmarks, and the samples from the 2 hemispheres were pooled. RNA was extracted using the RNeasy kit (Qiagen, 74104; Valencia, CA). Reverse transcription reactions were performed using the SuperScript III cDNA synthesis kit (Life Technologies, Grand Island, NY; 18080–044). Primer sequences for 18s (Life Technologies, Hs Hs99999901_s1) and dopamine receptors were chosen based on previously published data D1R (Life Technologies, Mm01353211_m1), D2R (Mm00438541_m1), D3R (Mm00432887_m1), D4R (Mm00432893_m1), and D5R (Mm00658653_s1). Real-time qPCR was performed in a StepOne Plus Thermocycler (Life Technologies) using Taqman PCR Master Mix (Life Technologies; 4369016) through 50 PCR cycles (95°C for 30 s, 57°C for 60 s, 72°C for 90 s). Levels of mRNA were normalized to 18 s. Samples from 4 to 6 mice were used for each experiment.

### High-performance liquid chromatography

Tissue was collected as described above for mRNA analysis. The orbitofrontal cortex, medical prefrontal cortex, and dorsal striatum were microdissected based on anatomical landmarks, and samples of each region were collected as described earlier [82–84]. For each brain region, samples from the right and left hemispheres from the same subject were pooled into a single sample for that subject. Each pooled sample was weighed and immediately frozen with liquid nitrogen. The tissue samples were shipped to the Neurochemistry Core at Vanderbilt University, Nashville, Tennessee, where they were homogenized and the protein concentration in each sample evaluated. Tissue concentrations (ng/mg protein) of dopamine and norepinephrine (NE) and their metabolites—DOPAC, HVA, and 3-MT—were analyzed.

### Collection of spermatozoa

Mice were euthanized by anesthetic overdose, and the testes were removed. Mature spermatozoa were collected using a double swim assay. Briefly, longitudinal cuts were made along the cauda epididymis in phosphate-buffered saline (pH 7.2) containing 1% bovine serum albumin to release the spermatozoa. The saline solution containing the spermatozoa was incubated at 37°C for 30 min, during which time the sperm swam up and collected in the supernatant. The supernatant was removed and incubated for an additional 10 min (“double swim assay”). A smear from each pellet was examined in a light microscope (Axiovert 25, Zeiss, Carl Zeiss, Thornwood, NY) to confirm the presence of only mature spermatozoa. Following cell counts, the spermatozoa were pelleted and frozen on dry ice and stored at −80°C until further use.

### DNA methylation and methylated DNA immunoprecipitation

Genomic DNA was isolated from pelleted sperm using a ZR Genomic DNA-Tissue Microprep kit (Zymo ZRGenomic; Zymo Research, Irvine, CA; D3041). Following isolation, DNA was eluted with 20 μl DNA elution buffer and quantified using a Qubit 2.0 fluorometer (Invitrogen, Carlsbad, CA). Next, 2.5 μl of normalized genomic DNA was amplified using a Qiagen REPLI-g Whole Genome Amplification kit (Qiagen, Valencia, CA; 150023) according to the manufacturer’s protocol. Typical DNA yields were greater than 4 μg for each sample. Amplified DNA was then prepared for anti-5-methylcytosine immunoprecipitation as follows: 2 μg of the DNA was diluted up to 50 μl using Zymo MIP buffer, vortexed, incubated on ice for 10 min, then subjected to fragmentation using a Diagenode Bioruptor 300 (30 s on; 90 s off; 7 cycles) to generate 200- to 500-bp fragments of genomic DNA. The amount of 1 μg of the disrupted DNA was stored as “input” DNA for later analysis. The remaining 1 μg of disrupted DNA was immunoprecipitated according to manufacturer’s protocol (Methylated-DNA IP Kit; Zymo Research, Irvine, CA; D5101) using a 1:10 μg ratio of DNA/anti-5-methylcytosine antibody. Negative controls included no antibody mock and IgG. The recovered DNA was quantified using a Qubit 2.0 fluorometer. Random priming amplification of immunoprecipitated DNA was carried out using an Affymetrix 2.0 DNA polymerase following the method of Cheung and colleagues [85] (Thermo Fisher Scientific; 70775X1000UN) and quantified using a Qubit 2.0 fluorometer.

Real-time thermal cycling was performed using methylated DNA immunoprecipitation (MeDIP)-derived DNA (2.5 ng/μL), 2X PerfeCTa SYBR Green SuperMix (QuantaBio, Beverly, MA; 95056–500), 0.5 μM of each primer, and StepOne Plus Thermocycler (Life Technologies). All PCR reactions were performed in triplicate. Target DNA sequence quantities were estimated from threshold amplification cycle numbers (Tc). For every gene sequence studied, a ΔTc value was calculated for each sample by subtracting the Tc value for Tc value for the input DNA (5 ng/μL) from the Tc value for the corresponding immunoprecipitated sample to normalize for differences in MeDIP sample aliquots. DNA quantities were expressed as percentages of corresponding input using the following equation: (antibody ChIP as a percentage of input) = 2 − (ΔTc) × 100.

Dopamine receptor primers were designed using NCBI and Methylprimer software The sequences for each are as follows: DRD1 forward GGT GCT GAA GAT TGA AGA TCC A; DRD1 reverse CGT CCT GAC ACA TGC TGT TAT AG; DRD2 forward ACC TGT CCT GGT ACG ATG ATG; DRD2 reverse GCA TGG CAT AGT AGT TGT AGT GG; DRD3 forward CCT CTG AGC CAG ATA AGC AGC; reverse AGA CCG TTG CCA AAG ATG ATG; DRD4 forward GCC TGG AGA ACC GAG ACT ATG; DRD4 reverse CGG CTG TGA AGT TTG GTG TG; DRD5 forward CTC GGC AAC GTC CTA GTG TG; and DRD5 reverse AAT GCC ACG AAG AGG TCT GAG.

### Statistical analysis

Main effects of paternal treatment, sex, and paternal treatment × sex interaction were analyzed using a two-way ANOVA. Acquisition learning on the Barnes maze (AL1-10; Fi. 1 E, F) was analyzed using a repeated-measures ANOVA. Post hoc pair-wise comparisons were performed using Bonferroni multiple comparisons test (Figs 1 and 2) when either of the 2 main factors (paternal treatment and sex) or the interaction between the factors was statistically significant by ANOVA. Each group was compared to every other group. For the F2 generation (Fig 3), following the ANOVA, a Dunnett test was used [22], as multiple comparisons were made against a single control group (F2 male or female mice from male or female F1 mice derived from plain drinking water–exposed F0 male founder; Fig 3). A two-tailed Student *t* test was used when differences between only 2 groups were evaluated (Fig 2K and 2L). GraphPad Prism 7.02 was used for all statistical analysis. The number of mice in each experimental group for each study is indicated in the legend to each Figure.

## Supporting information

S1 Table Tissue content of monoamines and their metabolites in the orbitofrontal cortex, medial prefrontal cortex, and striatum in the F1 generation (F1 water male *n* = 5–6; F1 nicotine male *n* = 6; F1 water female *n* = 6; F1 female nicotine *n* = 5–6). (XLSX)

S2 Table Dopamine receptor mRNA expression in the frontal cortex of the F1 generation (F1 water male *n* = 3–4; F1 nicotine male *n* = 4–5; F1 water female *n* = 3–4; F1 female nicotine *n* = 3–4). (XLSX)

S1 Fig (A) Spontaneous locomotor activity, (B) object-based attention, and (C) spatial working memory (Y-maze) in the F2 generation. (F2 water male *n* = 11–13; F2 female-derived nicotine male *n* = 11–13; F2 male-derived nicotine male *n* = 8–12; F2 water female *n* = 9–17, F2 female-derived nicotine female *n* = 8–13; F2 male-derived nicotine female *n* = 9–12; S3 Data).
(TIF)

S2 Fig (A) Spontaneous locomotor activity, (B) object-based attention, and (C) spatial working memory (Y-maze) in the F0 generation (water *n* = 8; nicotine *n* = 8–12; S5 Data).
(TIF)

S1 Data Data underlying Fig 1. (XLSX)

S2 Data Data underling Fig 2. (XLSX)

S3 Data Data underlying S1 Fig. (XLSX)

S4 Data Data underlying Fig 3. (XLSX)

S5 Data Data underlying S2 Fig. (XLSX)

## Abbreviations

3-MT: 3-methoxytyramine
ADHD: attention deficit hyperactivity disorder
DOPAC: 3,4-dihydroxyphenylacetic acid
HVA: homovanillic acid
MeDIP: methylated DNA immunoprecipitation
miRNA: microRNA
NE: norepinephrine
P0: postnatal day 0
qPCR: quantitative PCR
