# Supplementary figures and images for: Nicotine exposure of male mice produces behavioral impairment in multiple generations of descendants
Source: PLoS Biol. 2018 Oct 16;16(10):e2006497. doi: 10.1371/journal.pbio.2006497 (PMC6191076; doi:10.1371/journal.pbio.2006497)

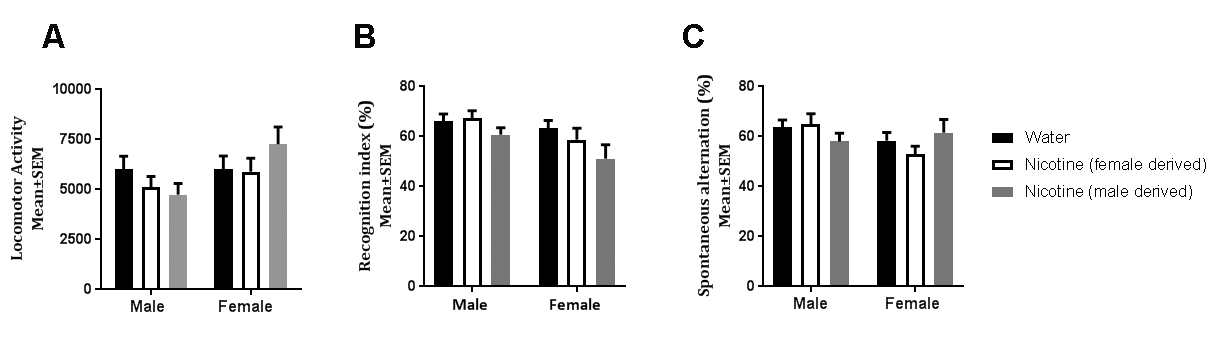

Supplement: S1 Fig — (A) Spontaneous locomotor activity, (B) object-based attention, and (C) spatial working memory (Y-maze) in the F2 generation. (F2 water male n = 11–13; F2 female-derived nicotine male n = 11–13; F2 male-derived nicotine male n = 8–12; F2 water female n = 9–17, F2 female-derived nicotine female n = 8–13; F2 male-derived nicotine female n = 9–12; S3 Data). (TIF) [file pbio.2006497.s003.tif]

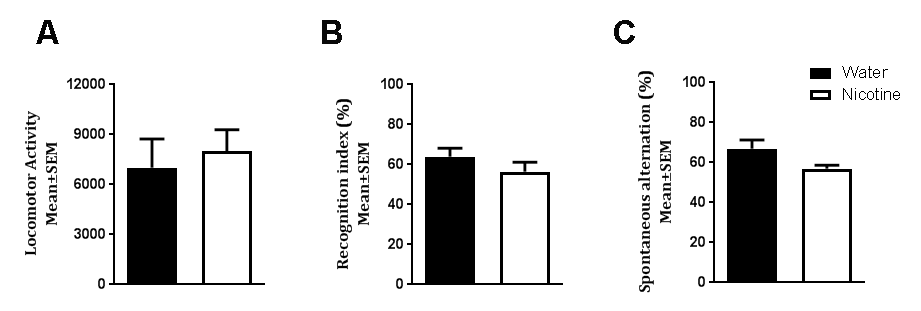

Supplement: S2 Fig — (A) Spontaneous locomotor activity, (B) object-based attention, and (C) spatial working memory (Y-maze) in the F0 generation (water n = 8; nicotine n = 8–12; S5 Data). (TIF) [file pbio.2006497.s004.tif]
